# Preparation of a Thermosensitive Chitosan–Sea Cucumber Peptide Hydrogel and Its Alleviating Effect on Acute Alcohol-Induced Dual Liver and Brain Injury in Mice

**DOI:** 10.3390/md24090294

**Published:** 2026-08-23

**Authors:** Jiaqi Guo, Songzhi Kong, Chen Chen, Guiping Lu, Zirui Li, Jinhui Chen, Meiyin Liang

**Affiliations:** 1 College of Chemistry and Environment, Guangdong Ocean University, Zhanjiang 524088, China; amieshuigjq@163.com (J.G.); 15916290483@163.com (C.C.); 18216727176@163.com (G.L.); l100353641@163.com (Z.L.); 15768623280@139.com (J.C.); 13242102212@163.com (M.L.); 2 Faculty of Medicine, Macao University of Science and Technology, Macao 999078, China

**Keywords:** sea cucumber peptides, chitosan, thermosensitive hydrogel, alcoholic liver injury, alcoholic brain injury

## Abstract

Excessive short-term ethanol intake often causes acute intoxication and multi-organ damage, especially to the liver and brain. Thus, developing safe and effective preparations for hangover relief, liver protection, and brain function regulation is of great practical significance. In this study, we fabricated a thermosensitive chitosan (CS)–sea cucumber peptide (SCP) hydrogel (CS–SCP gel) loaded with SCP using NaHCO_3_ as a crosslinker and characterized its properties. Kunming mouse models of anti-intoxication and acute alcohol-induced liver and brain injuries were established. The anti-intoxication and organ-protective effects of the CS–SCP gel were comprehensively evaluated through behavioral observation, liver histopathology, and biochemical assays of serum and liver, kidney, and brain tissues. The CS–SCP gel exhibited a phase transition temperature of 35.7 °C, a water absorption rate of 1015.47%, and a cumulative peptide release of 74.83% within 330 min. In mice, it prolonged the latency to drunkenness; shortened alcohol-induced sleep and sobering time; reduced blood ethanol, transaminase, and lipid levels; upregulated hepatic antioxidant enzymes; downregulated pro-inflammatory cytokines, and alleviated lipid peroxidation. It also enhanced brain antioxidant capacity, suppressed cerebral inflammatory cytokines, and maintained cholinergic neurotransmitter homeostasis. Collectively, with sustained release, CS–SCP gel is expected to prolong the pharmacological action of SCP, enhance therapeutic efficacy, and protect against alcohol-induced liver and brain injury through the regulation of oxidative stress and attenuation of inflammation.

## 1. Introduction

With rapid social and economic development, the production and consumption of alcoholic beverages have been increasing year by year, accompanied by a corresponding rise in both the number of alcohol users and total alcohol consumption. According to the WHO Global Status Report on Alcohol and Health and Treatment of Substance Use Disorders, an estimated 400 million people worldwide live with alcohol use disorders, of whom 209 million have alcohol dependence [1]. Excessive short-term alcohol consumption can lead to acute alcohol intoxication (commonly known as drunkenness), which is clinically classified into three stages—excitement, ataxia, and coma; however, the associated risk of organ damage is often overlooked by the general public [2]. When the amount of ethanol ingested exceeds the liver’s metabolic capacity, ethanol and its metabolite acetaldehyde accumulate in the liver, causing oxidative stress and inflammatory responses that lead to liver damage [3]. Meanwhile, excess ethanol and acetaldehyde enter the brain through the blood circulation, disrupting central neurotransmission and triggering symptoms such as gait imbalance, drowsiness, and headache; in severe cases, convulsions, respiratory depression, and even death may occur [4]. Therefore, there is a strong practical demand for the development of safe and effective functional preparations that can relieve hangovers and counteract alcohol-induced liver and brain injury.

In recent years, compared with chemically synthesized drugs, natural bioactive substances with low toxicity and good biocompatibility have become a research focus in functional products for hangover relief and organ protection. Sea cucumbers, being widely distributed, protein-rich and cost-effective, possess considerable potential for further development and application. Sea cucumber peptides (SCPs) are functional bioactive peptides extracted from sea cucumbers and have been demonstrated to possess excellent antioxidant, anti-inflammatory and immunomodulatory activities [5,6]. Meanwhile, previous studies have demonstrated that sea cucumber peptides exert protective effects against alcoholic liver injury [7,8,9]. These findings suggest that the potential of SCPs in hangover relief, liver protection, and neuroprotection is worth further exploration. However, like most bioactive peptides, SCPs suffer from a short in vivo half-life, low permeability, poor bioavailability, and limited in vivo stability [10]. Therefore, employing appropriate biological carriers to encapsulate and protect SCP, thereby prolonging its duration of action, is of great research significance for fully realizing the application value of sea cucumber peptides in hangover relief and organ protection.

Chitosan (CS) is a natural polysaccharide extracted from shrimp and crab shells. It is the amino-containing cationic alkaline polysaccharide in nature and possesses excellent biocompatibility, mucoadhesiveness, biodegradability, and low immunogenicity, as well as antioxidant and anti-inflammatory activities [11,12]. As a natural polymer, CS also exhibits unique gel-forming properties. Its molecular chains, rich in amino and hydroxyl groups, readily form intra- and intermolecular hydrogen bonds, enabling the construction of hydrogels with porous three-dimensional networks through physical or chemical crosslinking [13]. CS-based hydrogels exhibit excellent water absorption, ethanol adsorption, and sustained-release properties, making them excellent delivery carriers for protein- and gene-based therapeutics [14]. The introduction of thermosensitive polymers into chitosan hydrogels can impart thermo-responsive properties [15]. Thermosensitive gels can undergo gelation at physiological temperature. The underlying gelation mechanism is based on temperature-induced changes in intermolecular forces, which trigger a phase transition of the polymer material from a liquid to a semi-solid state [16]. The polymers are expected to delay alcohol absorption while continuously exerting organ-protective and reparative effects, thus holding considerable research and application value in the prevention and treatment of alcoholic liver and brain injury.

Based on this, the present study utilized natural marine polysaccharide CS as the matrix and NaHCO_3_ as the crosslinker to encapsulate SCP, fabricating a CS–SCP gel. After oral administration, since NaHCO_3_ is a weak base, HCO_3_^−^ reacts with H^+^ to generate carbon dioxide (CO_2_). Within the [CS-NH_3_^+^] [HCO_3_^−^] weak acid–weak base equilibrium system, this process promotes the conversion of protonated amino groups (CS-NH_3_^+^) to deprotonated amino groups (CS-NH_2_), thereby inducing the crosslinking and entanglement of chitosan molecular chains and enabling an in situ sol–gel phase transition at physiological temperature [17]. Based on the physicochemical properties above, this thermosensitive gel can theoretically intervene in alcohol-induced systemic injury through multiple pathways, including physical barrier formation, ethanol retention, and sustained release of active peptides.

## 2. Results

### 2.1. Molecular Weight Characteristics of SCP

**Figure 1:**
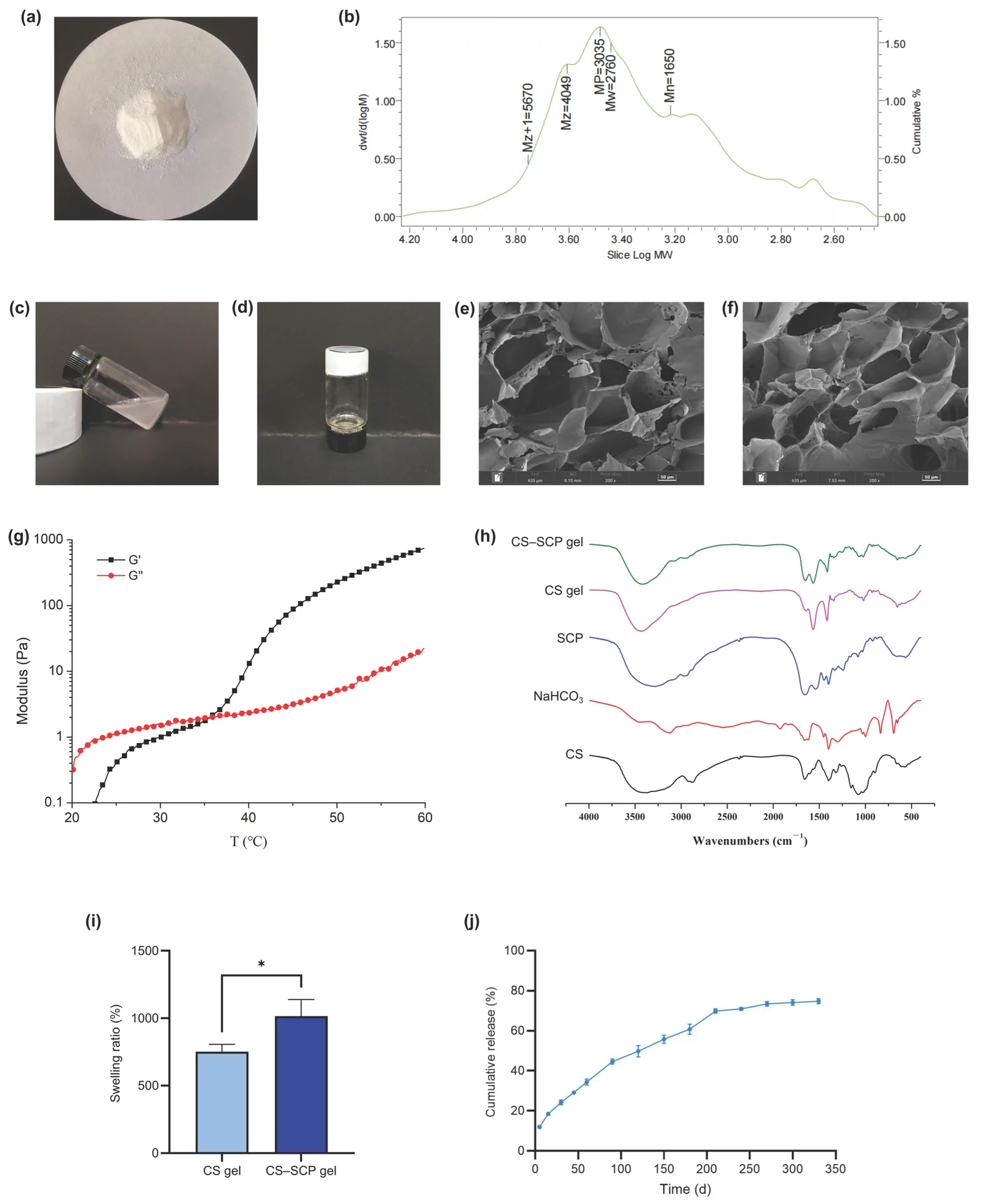
(**a**) Macroscopic appearance of SCP. (**b**) The molecular weight distribution of SCP. (**c**) Images of the temperature-sensitive sol states. (**d**) Images of the gel states. (**e**) The electronic spectra of CS gel. (**f**) The electronic spectra of CS–SCP gel. (**g**) Storage modulus (G′) and loss modulus (G″) of CS–SCP gel as a function of temperature. (**h**) FTIR spectra of CS–SCP gel, CS gel, SCP, NaHCO_3_ and CS. (**i**) Water absorption rates of CS–SCP gel and CS gel. Cumulative drug release rate of CS–SCP gel (**j**). * *p* < 0.05.

The SCP was a white, dry powder (Figure 1a). As shown in Table 1 and Figure 1b, the molecular weight distribution curve of SCP exhibited a prominent single peak, indicating a relatively concentrated molecular weight distribution. The molecular weight was predominantly below 4000 Da, with the 2000–3000 Da fraction accounting for the highest proportion at 32.52%, and the fraction below 2000 Da accounting for 25.60%. Together, these two fractions accounted for 58.12%, suggesting that the majority of SCP had molecular weights in the range of 2000–3000 Da. Furthermore, the fraction below 4000 Da reached 84.82%, indicating that although SCP is a mixture of peptides with different molecular weights, the main components are of relatively low molecular weight. Small-molecule therapeutic agents (<500 Da) typically undergo rapid release from hydrogels, whereas larger therapeutic agents (>10 kDa) diffuse slowly. Excessively high drug concentrations may induce toxicity, while insufficient plasma drug levels fail to achieve the desired therapeutic effect [18]. With a molecular weight of 2000–3000 Da, SCP is well-matched to the three-dimensional network of chitosan hydrogels.

### 2.2. The Amino Acid Composition of SCP

The radical-scavenging activity of peptides is closely related to their amino acid composition: hydrophobic amino acids can donate protons and inhibit lipid oxidative damage, while acidic amino acids possess both free radical-scavenging and metal ion-chelating abilities, thereby attenuating metal-catalyzed oxidative effects and protecting against lipid peroxidation [19]. As shown in Table 2, the predominant amino acids in SCP were Gly, Pro, Glu, and Ala, accounting for 16.189%, 11.506%, 11.043%, and 9.285%, respectively. Among them, hydrophobic amino acids (Phe, Val, Leu, Ile, Ala, Pro, and Met) and acidic amino acids (Asp and Glu) accounted for 39.343% and 19.496%, respectively. Park et al. [20] isolated a peptide (KPF) from krill with a molecular weight of 1–3 kDa, rich in Glu, Asp, Leu, Lys, and Arg. Animal experiments demonstrated that KPF could regulate the levels of superoxide dismutase (SOD), Catalase (CAT), and Glutathione Peroxidase (GSH-Px) in mouse liver tissue, while decreasing the mRNA expression of tumor necrosis factor-α (TNF-α) and interleukin-6 (IL-6), upregulating Nrf2 and HO-1 expression, and inhibiting ethanol-induced hepatic apoptosis protein production, thereby exerting a protective effect against acute alcoholic liver injury. It can thus be inferred that low-molecular-weight SCP may effectively scavenge free radicals and inhibit lipid peroxidation, suggesting that its potential in the prevention and treatment of acute alcoholic liver and brain injury warrants further exploration.

### 2.3. Macroscopic Morphology and Microstructure (SEM) Analysis of the Hydrogels

At room temperature, the chitosan–sea cucumber peptide sol was a milky white, flowable liquid (Figure 1c). When the temperature reached ≥37 °C, a pronounced sol–gel phase transition occurred, and the liquid visibly transformed into a jelly-like solid hydrogel, namely, CS–SCP gel (Figure 1d).

The microstructures of the CS gel and CS–SCP gel were observed by SEM (Figure 1e,f). Both hydrogels exhibited a three-dimensional porous network structure. Compared with CS gel, the CS–SCP gel showed more abundant pores and a denser pore size distribution. This porous network not only facilitates the absorption of excess ethanol and swelling of the hydrogel in the stomach, forming a physical protective barrier on the gastric mucosal surface that shields against direct irritation and damage from high-concentration ethanol [21], but also provides interconnected channels for the diffusion and release of SCP, highlighting the excellent potential of this thermosensitive hydrogel for drug delivery applications.

### 2.4. Gelling Temperature of CS–SCP Gel

At room temperature, the chitosan–sea cucumber peptide sol exhibited good fluidity and existed as a homogeneous liquid system. It could undergo a phase transition and gelation in the temperature range of 35–37 °C, forming CS–SCP gel. The rheological curve (Figure 1g) showed that the crossover point of the storage modulus (G′) and loss modulus (G″) occurred at 35.7 °C, which was identified as the sol–gel transition temperature of the CS–SCP sol. When the temperature exceeded 35.7 °C, the system completely transformed from the sol state to the gel state. Since the normal human body temperature is 36–37 °C, this sol can successfully undergo in situ sol–gel transition under the physiological temperature conditions of the gastrointestinal tract.

### 2.5. Comparative Analysis of Infrared Absorption Spectra of Test Samples

The FTIR spectra of the samples are shown in Figure 1h. Compared with CS, both the CS–SCP gel and CS gel exhibited a characteristic absorption peak near 1564 cm^−1^, attributed to the N–H bending vibration of the amide II band. This was presumably due to the conversion of CS-NH_3_^+^ to CS-NH_2_ under the pH regulation of NaHCO_3_, indicating that the hydrogel was formed through physical crosslinking. In addition, the –C–H stretching vibration peak of CS around 2877 cm^−1^ disappeared in the spectra of both hydrogels, suggesting the formation of intermolecular hydrogen bonds between CS and NaHCO_3_. A comparison of the FTIR spectra of the CS–SCP gel and CS gel revealed that the positions and profiles of their characteristic peaks were essentially identical, indicating that SCP was mainly incorporated into the hydrogel network through physical blending and that no chemical reaction occurred during the loading process.

### 2.6. Water Absorption Rate

The water absorption rates of the CS gel and CS–SCP gel are shown in Figure 1i. Both freeze-dried hydrogels exhibited water absorption rates above 700%. After SCP loading, the water absorption rate of the CS–SCP gel further increased, reaching 1015.47%. This indicates that the incorporation of SCP did not disrupt the porous structure of the hydrogel. Instead, it optimized the pore architecture and enhanced the water absorption and swelling capacity. The results above indicate that CS–SCP gel exhibits excellent water absorption and swelling properties. We speculate that CS–SCP gel may delay the time to peak blood ethanol concentration by retaining ethanol in the stomach, thereby reducing hepatic metabolic burden and ethanol accumulation in the brain. This may be one of the potential mechanisms.

### 2.7. In Vitro Release Profile of SCP from CS–SCP Gel

As shown in Figure 1j, the release rate of SCP was fastest during the first 90 min, exhibiting a slight burst release effect. Between 90 and 210 min, the release rate decelerated, and the drug was released in a steady and sustained manner. In the later stage, the release process became gradual and prolonged, with a cumulative SCP release rate of 74.83% at 330 min. These results indicate that CS–SCP gel can achieve the phased and long-term sustained release of SCP, which is conducive to prolonging the retention time of the peptide in vivo after administration.

### 2.8. Optimal Intoxicating Alcohol Dose in Mice

**Figure 2:**
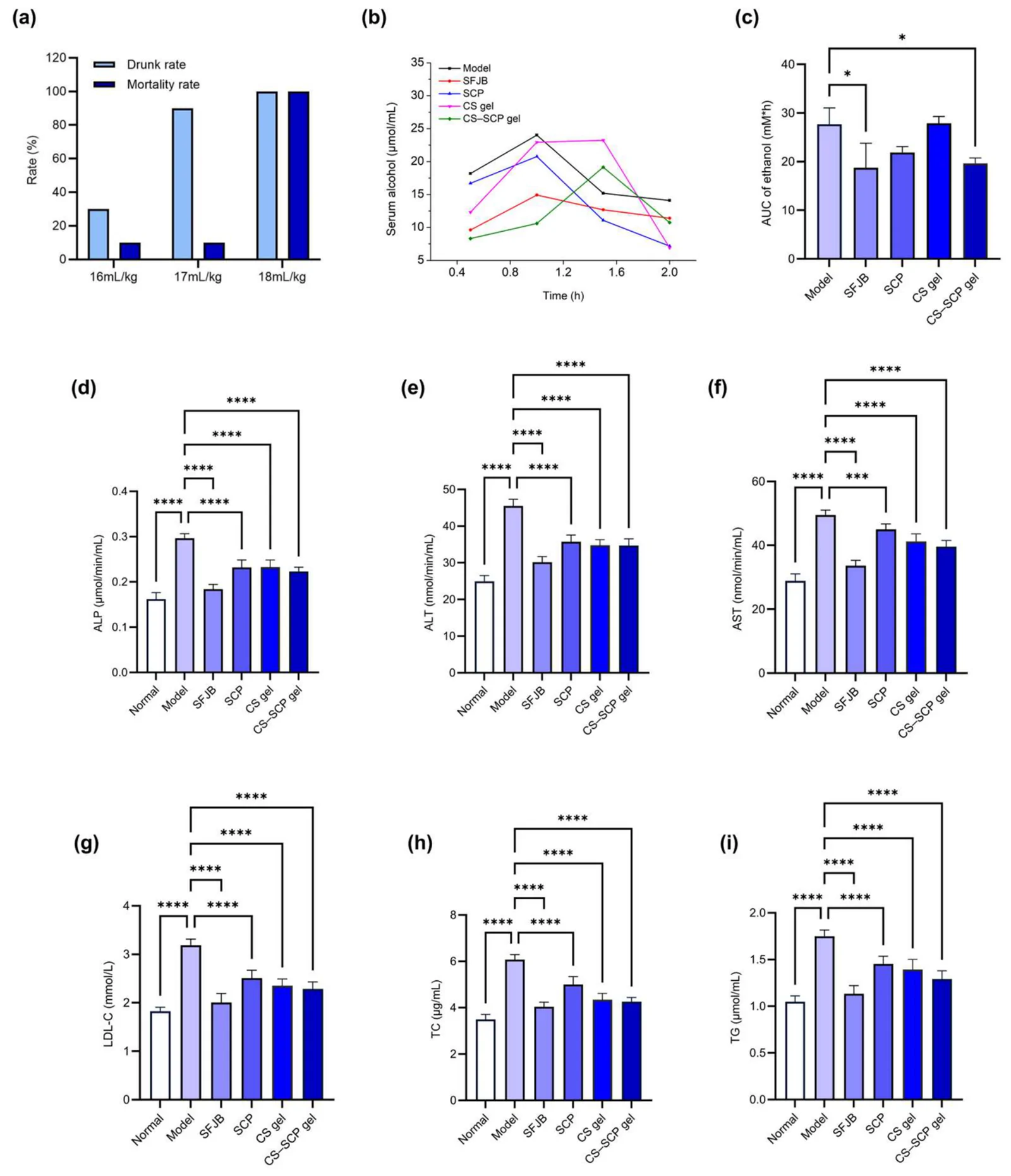
(**a**) Drunkenness rate and mortality rate of different alcohol doses (*n* = 10). (**b**) The concentration of ethanol in the serum of mice in each group (*n* = 3). (**c**) The AUC for serum ethanol (*n* = 3). Levels of (**d**) ALP, (**e**) ALT, (**f**) AST, (**g**) LDL-C, (**h**) TC and (**i**) TG in serum (*n* = 6). Data are shown as mean ± SD ($\overset{-}{x}$ ± SD). * *p* < 0.05 vs. Model group, *** *p* < 0.001 vs. Model group, **** *p* < 0.0001 vs. Model group.

As shown in Figure 2a, a gavage-administered dose of alcohol of 16 mL/kg resulted in a 10% mortality rate, while the incidence of intoxication was insufficient to satisfy the modeling criteria. Upon increasing the dosage to 17 mL/kg, the intoxication incidence rose to 90% alongside a 10% mortality rate. This condition allowed successful construction of the acute-intoxication model with relatively low risks of severe damage to the experimental animals. At 18 mL/kg, both the intoxication rate and mortality rate reached 100%, indicating excessive toxicity and poor model stability. Taken together, 17 mL/kg was ultimately determined as the optimal intoxicating alcohol gavage dose in mice.

### 2.9. Anti-Intoxication Efficacy in Mice

As shown in Table 3, the intoxication and mortality rates in the Model group were as high as 91.67% and 50%, respectively. Following intervention, both rates decreased across all the treatment groups: SFJB (83.33%, 41.67%), SCP (75.00%, 16.67%), CS gel (75.00%, 8%), and CS–SCP gel (58.33%, 0%). Among them, the CS–SCP gel exhibited the most pronounced effect in reducing the risk of intoxication and death. Compared with the Model group, SFJB, SCP and CS–SCP gel all prolonged the latency to intoxication. Regarding sleep maintenance time, SFJB, SCP, CS gel, and CS–SCP gel all shortened the duration of alcohol-induced sleep in mice to varying degrees, with the CS–SCP gel group showing a statistically significant difference (*p* < 0.01). Meanwhile, CS–SCP gel also shortened the sobering time in mice, showing a highly significant difference compared with the Model group. (*p* < 0.01). In summary, CS–SCP gel demonstrated superior overall anti-intoxication efficacy compared with free SCP and the positive control drug SFJB, exhibiting the best anti-intoxication effect.

### 2.10. Blood Ethanol Concentration Changes in Mice

The changes in blood ethanol concentration at different time points in each group of mice are shown in Figure 2b. In the Model, SFJB, and SCP groups, blood ethanol concentrations increased continuously from 0 to 1.0 h after gavage, peaked at 1.0 h, and then gradually declined. In the CS gel and CS–SCP gel groups, the ascending phase of blood ethanol concentration was extended to 1.5 h, with concentrations beginning to decline only after 1.5 h. Compared with the Model group, both CS gel and CS–SCP gel delayed the time to peak blood ethanol concentration and slowed the absorption of ethanol into the bloodstream, and CS–SCP gel significantly reduced the peak ethanol concentration. The area under the curve (AUC) of serum ethanol is shown in Figure 2c. Compared with the Model group, treatment with CS–SCP gel reduced the AUC of serum ethanol by 8.04%, with a statistically significant difference. We speculate that ethanol retention in the stomach could be one potential mechanism by which CS–SCP gel and CS gel delay the time-to-peak blood ethanol concentration.

### 2.11. Effect of CS–SCP Gel on Liver Function and Blood Lipid Levels in Mice with Acute Alcohol-Induced Injury

As shown in Figure 2d–f, compared with the Normal group, the Model group showed significantly elevated serum activities of alkaline phosphatase (ALP), alanine aminotransferase (ALT), and aspartate aminotransferase (AST) (all *p* < 0.0001), indicating that a single excessive alcohol intake can directly damage hepatocytes and induce acute liver injury. Compared with the Model group, interventions with SFJB, SCP, CS gel, and CS–SCP gel all significantly reduced the serum activities of ALT, AST, and ALP. Among them, the CS gel and CS–SCP gel groups showed marked improvement (*p* < 0.0001), with efficacy comparable to that of the SFJB group. Furthermore, the levels of these three indicators in the CS–SCP gel group were even lower than those in the SCP group and the CS gel group. These results confirm that CS–SCP gel can effectively suppress alcohol-induced hepatic enzyme abnormalities and protect damaged hepatocytes.

As shown in Figure 2g–i, the serum levels of low-density lipoprotein cholesterol (LDL-C), total cholesterol (TC), and triglycerides (TG) in the Model group were significantly elevated compared with the Normal group (all *p* < 0.0001), indicating that acute alcohol exposure disrupts hepatic lipid metabolism and leads to dyslipidemia. Compared with the Model group, all the treatment groups were able to downregulate the abnormal blood lipid levels. Among them, CS gel and CS–SCP gel exhibited highly significant regulatory effects (*p* < 0.0001), and the effect of CS–SCP gel was comparable to that of the positive control drug SFJB. Moreover, the CS–SCP gel demonstrated greater improvement in blood lipid parameters compared with SCP alone and CS gel, suggesting a synergistic effect between the chitosan carrier and SCP, which effectively alleviates alcohol-induced hepatic lipid metabolism disorders.

### 2.12. Effect of CS–SCP Gel on Hepatic Oxidative Stress and Inflammatory Pathways in Mice with Acute Alcohol-Induced Injury

**Figure 3:**
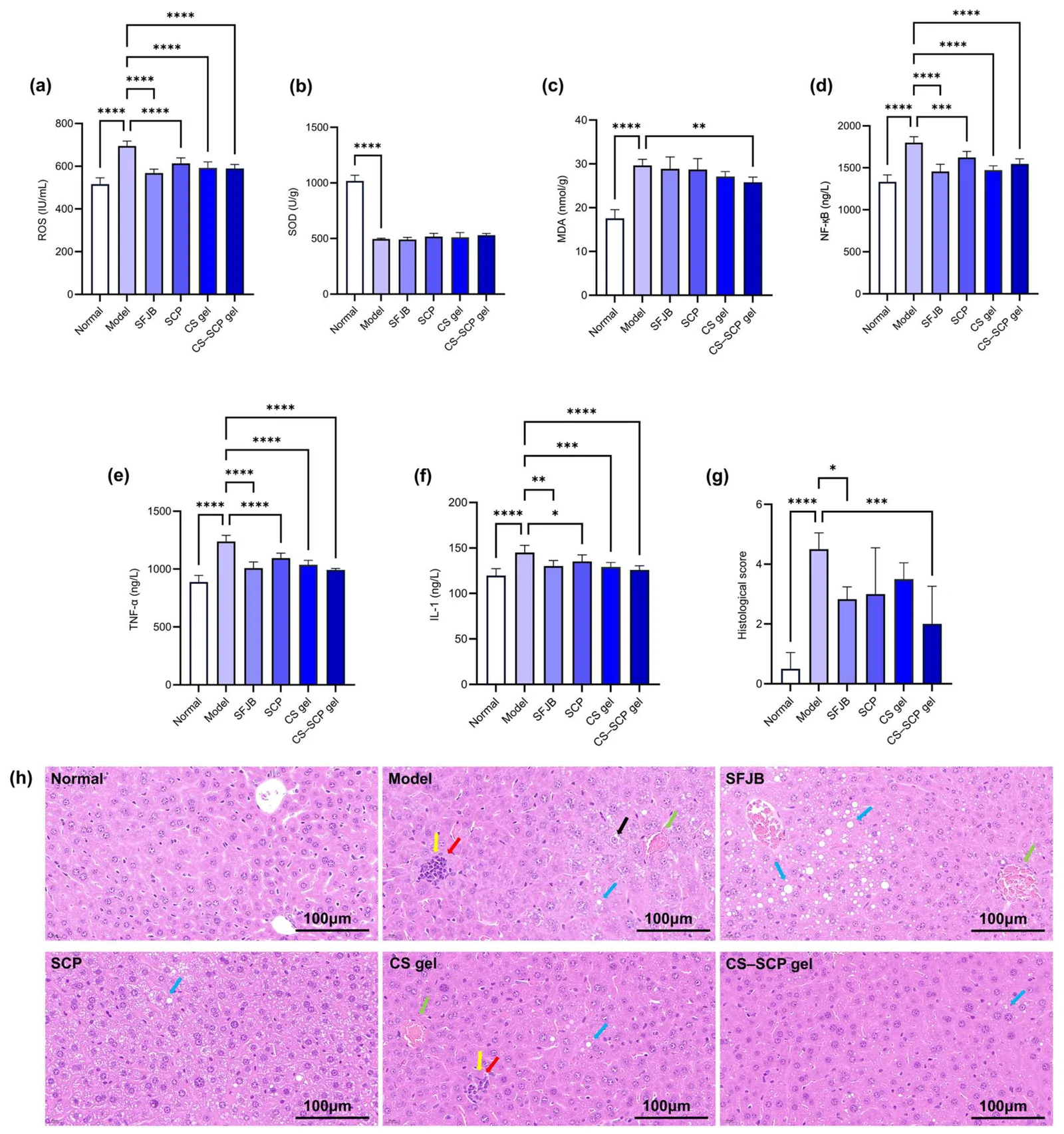
(**a**) Levels of ROS, (**b**) SOD, (**c**) MDA, (**d**) NF-κB, (**e**) TNF-α and (**f**) IL-1 in liver of mice. (**g**) Histological scores based on the histological slides from (**h**). (**h**) HE staining to evaluate the pathological changes of liver tissue in each group (400×). Data are shown as mean ± SD ($\overset{-}{x}$ ± SD, *n* = 6). * *p* < 0.05 vs. Model group, ** *p* < 0.01 vs. Model group, *** *p* < 0.001 vs. Model group, **** *p* < 0.0001 vs. Model group. Notes: The black arrow indicates watery degeneration of mouse hepatocytes, the blue arrow indicates mild steatosis of mouse hepatocytes, the red arrow indicates focal necrosis of mouse hepatocytes, the yellow arrows indicate lymphocyte aggregation, and the green arrows indicate vascular congestion in mouse-liver tissue.

As shown in Figure 3a–c, compared with the Normal group, the Model group exhibited significantly elevated hepatic reactive oxygen species (ROS) and malondialdehyde (MDA) levels, along with markedly reduced SOD activity (all *p* < 0.0001), indicating that acute alcohol intake can trigger hepatic lipid peroxidation and oxidative stress injury. Compared with the Model group, administration of SFJB, SCP, CS gel, and CS–SCP gel all ameliorated the oxidative abnormalities above to varying degrees. Notably, CS–SCP gel significantly reduced ROS levels (*p* < 0.0001) and downregulated MDA (*p* < 0.01), and its overall antioxidant capacity was superior to that of the positive control drug SFJB. These findings suggest that CS–SCP gel can scavenge hepatic free radicals, inhibit lipid peroxidation, and alleviate alcohol-induced oxidative damage in hepatocytes.

As shown in Figure 3d, the expression of nuclear factor-κB (NF-κB) in the liver tissue of the Model group was significantly higher than that of the Normal group (*p* < 0.0001), suggesting that acute alcohol exposure elevates NF-κB levels and exacerbates hepatic inflammatory injury. Compared with the Model group, all the treatment groups downregulated NF-κB levels to varying degrees. Among them, SFJB, CS gel, and CS–SCP gel showed highly significant improvement (all *p* < 0.0001), and their effects were superior to that of SCP. Furthermore, as shown in Figure 3e,f, the levels of TNF-α and interleukin-1 (IL-1) in the Model group were markedly elevated compared with the Normal group (*p* < 0.0001), indicating that excessive ethanol triggers a strong hepatic inflammatory response. Compared with the Model group, all the tested substances reduced the levels of these two pro-inflammatory cytokines. CS–SCP gel exhibited a stronger inhibitory effect than SCP and SFJB and was slightly superior to CS gel. In summary, CS–SCP gel can alleviate hepatic inflammatory injury induced by acute alcohol exposure by downregulating the expression of pro-inflammatory cytokines, demonstrating a favorable hepatoprotective effect.

### 2.13. Hepatic Histopathology in Mice with Acute Alcohol-Induced Injury

The H&E-stained liver sections of mice from each group are shown in Figure 3h, and the corresponding semi-quantitative histological scores are presented in Figure 3g. In the Normal group, the hepatic lobule architecture was intact, hepatocytes were arranged in an orderly manner, and the cytoplasm and nuclei exhibited normal morphology, with no evident degeneration, necrosis, or inflammatory infiltration. Compared with the Normal group, the Model group showed marked hepatic tissue injury, characterized by abundant hepatocellular hydropic degeneration (black arrows) and steatosis (blue arrows), accompanied by focal hepatocellular necrosis (red arrows, featuring pyknosis, karyorrhexis, and karyolysis), vascular congestion (green arrows), and scattered lymphocyte aggregates (yellow arrows). The semi-quantitative histological score was significantly elevated (*p* < 0.0001), indicating that high-dose alcohol disrupts hepatic tissue architecture and induces steatosis and inflammation. All the interventions alleviated the hepatic pathological damage to varying degrees. Notably, in the CS–SCP gel group, the hepatic lobule architecture remained largely intact, hepatocytes were regularly arranged, and hydropic degeneration, steatosis, and inflammatory infiltration were markedly alleviated, with the semi-quantitative histological score significantly decreased (*p* < 0.001). In conclusion, CS–SCP gel effectively ameliorates alcohol-induced hepatocellular degeneration and inflammation.

### 2.14. Effect of CS–SCP Gel on the Antioxidant System and Inflammatory Cytokines in the Brain of Mice with Acute Alcohol-Induced Injury

**Figure 4:**
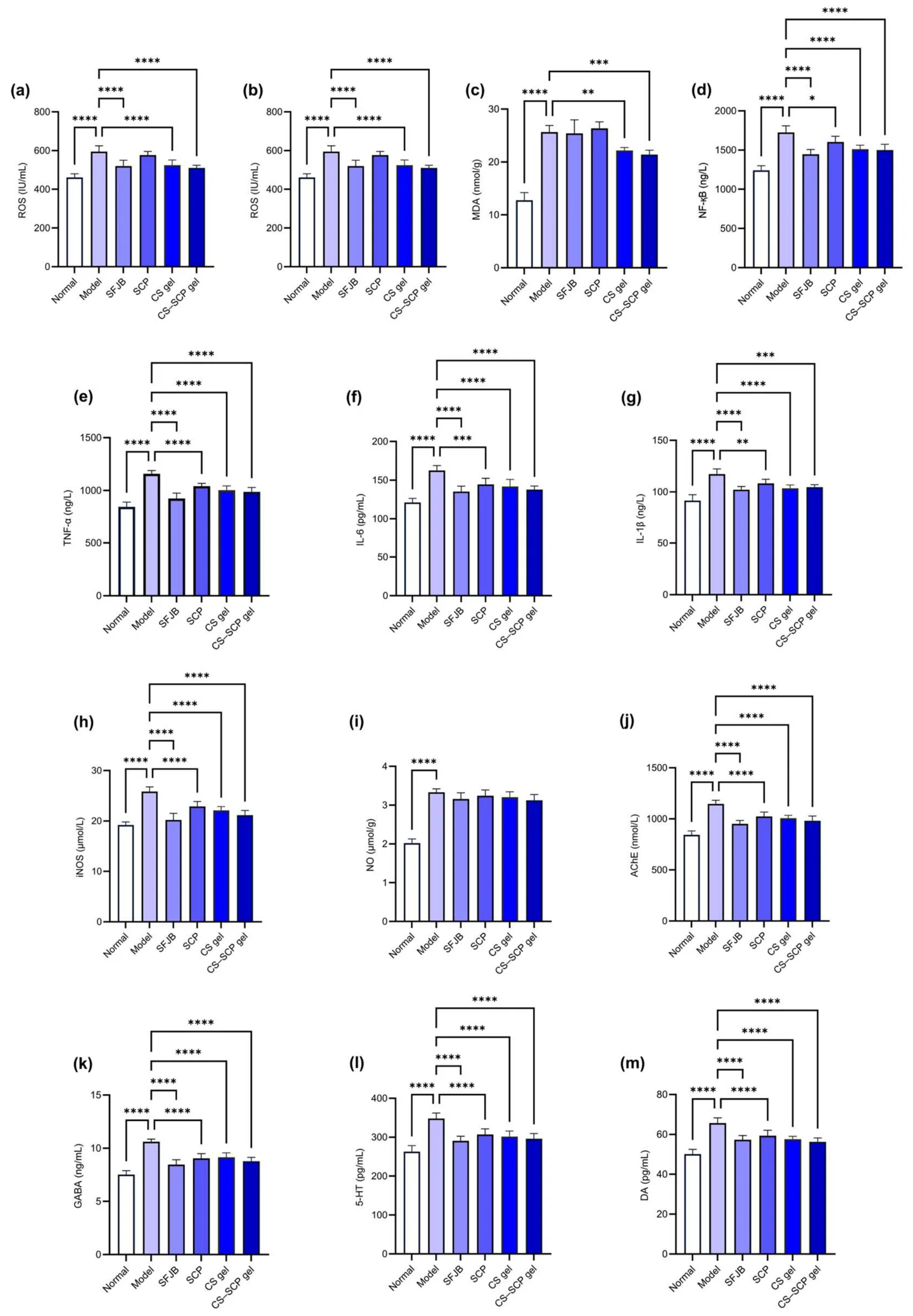
Levels of (**a**) ROS, (**b**) SOD, (**c**) MDA, (**d**) NF-κB, (**e**) TNF-α, (**f**) IL-6, (**g**) IL-1β, (**h**) iNOS, (**i**) NO, (**j**) AChE, (**k**) GABA, (**l**) 5-HT, and (**m**) DA in brain of mice. Data are shown as mean ± SD ($\overset{-}{x}$ ± SD, *n* = 6). * *p* < 0.05 vs. Model group, ** *p* < 0.01 vs. Model group, *** *p* < 0.001 vs. Model group, **** *p* < 0.0001 vs. Model group.

The results of brain tissue oxidative stress indicators are shown in Figure 4a–c. Compared with the Normal group, the Model group displayed markedly elevated cerebral ROS and MDA levels (*p* < 0.0001), whereas SOD activity was remarkably reduced (*p* < 0.0001). These findings demonstrate that excess ethanol triggered oxidative-stress responses and subsequent neuronal oxidative injury within brain tissue. Interventions with SFJB, SCP, CS gel, and CS–SCP gel all reduced brain ROS levels, increased the activity of the antioxidant enzyme SOD, and decreased the MDA content to varying degrees. Among them, CS–SCP gel showed the most pronounced effect, which was superior to that of SFJB. These results suggest that CS–SCP gel can effectively reverse the alcohol-induced decline in brain antioxidant capacity, scavenge excess free radicals, inhibit lipid peroxidation, and alleviate oxidative damage to neuronal cells.

Figure 4d presents the analysis of the NF-κB-related inflammatory pathway in brain tissues. The Model group exhibited an extremely high level of cerebral NF-κB relative to the Normal group (*p* < 0.0001). Compared with the Model group, all the tested substances downregulated NF-κB expression to varying degrees. Among them, CS–SCP gel exhibited a marked inhibitory effect (*p* < 0.0001), and its effect was superior to that of SCP.

The results of pro-inflammatory cytokine measurements in brain tissue are shown in Figure 4e–g. Compared with the Normal group, acute alcohol exposure significantly increased the levels of TNF-α, IL-6, and interleukin-1β (IL-1β) in the brain tissue of mice (all *p* < 0.0001), triggering severe neuroinflammation. Compared with the Model group, SCP significantly reduced the levels of these pro-inflammatory cytokines, while both CS gel and CS–SCP gel exerted marked inhibitory effects on all three indicators (*p* < 0.0001). Moreover, the regulatory effect of CS–SCP gel was superior to that of SCP alone and CS gel and was comparable to that of the positive control drug SFJB. These results indicate that CS–SCP gel can effectively inhibit alcohol-induced neuroinflammatory responses and significantly ameliorate brain inflammatory injury.

The levels of iNOS and NO in brain tissue are shown in Figure 4h,i. Compared with the Normal group, the Model group exhibited markedly elevated levels of inducible nitric oxide synthase (iNOS) and nitric oxide (NO) (*p* < 0.0001). These data reveal that ethanol stimulation facilitates iNOS production, triggers excessive NO accumulation, and ultimately induces neurotoxicity and neuronal injury. Compared with the Model group, SFJB, SCP, CS gel, and CS–SCP gel all significantly downregulated iNOS levels in brain tissue (all *p* < 0.0001) and reduced NO accumulation to varying degrees. Among them, CS–SCP gel demonstrated the best intervention effect, which was comparable to that of SFJB. In conclusion, CS–SCP gel exerts favorable protective effects on brain tissue by attenuating alcohol-mediated neurocytotoxicity.

### 2.15. Effect of CS–SCP Gel on Neurotransmitter Levels in the Brain of Mice with Acute Alcohol-Induced Injury

As shown in Figure 4j–m, the Model group displayed markedly higher acetylcholinesterase (AChE) activity, as well as increased contents of γ-aminobutyric acid (GABA), 5-hydroxytryptamine (5-HT) and dopamine (DA) in brain tissues relative to the Normal group (*p* < 0.0001). These results demonstrate that excessive ethanol intake breaks cerebral neurotransmitter homeostasis, impairs neuronal signal transduction, and further induces intoxication-associated manifestations, including gait imbalance and drowsiness. Compared with the Model group, interventions with SFJB, SCP, CS gel, and CS–SCP gel all effectively ameliorated the abnormally elevated AChE activity and neurotransmitter levels. Among them, the SFJB and CS–SCP gel groups showed the most pronounced and comparable effects. These results indicate that CS–SCP gel can effectively correcting alcohol-induced neurotransmitter imbalances, restoring central neurotransmission, and alleviating symptoms of intoxication.

## 3. Discussion

Acute excessive alcohol consumption can induce systemic oxidative stress, lipid metabolism disorders, and inflammatory cascades, leading to dual injuries to the liver and brain. Therefore, the development of safe and effective natural anti-hangover and brain-protective preparations is of great practical significance [22]. Multiple studies have demonstrated that chitosan-based hydrogels can load natural bioactive ingredients and protect body tissues through antioxidant, anti-inflammatory, and physical barrier mechanisms, while effectively prolonging drug efficacy and enhancing bioavailability [23,24,25,26]. Capitalizing on these excellent properties of chitosan hydrogels, a thermosensitive CS–SCP gel was successfully fabricated in this study. The material is safe and non-toxic, and its physicochemical characteristics are well-suited for oral anti-intoxication intervention. The hydrogel exhibited a sol–gel phase transition temperature of 35.7 °C, enabling in situ gelation at the physiological temperature of the gastrointestinal tract. Benefiting from its highly porous microstructure, the hydrogel attained a water-absorption capacity of 1015.47%. Owing to its excellent water absorption properties, this hydrogel is expected to physically retain ethanol in the stomach, thereby reducing the local free ethanol concentration and delaying ethanol absorption. This may be one of the potential mechanisms. The in vitro release results demonstrate that the CS–SCP gel achieved a sustained-release effect of SCP, with a cumulative release rate of 74.83% within 330 min, effectively compensating for the short duration of action of free SCP and enabling SCP to continuously exert organ-protective and reparative effects.

Acute alcohol exposure induces liver and brain injury through pathways such as oxidative stress, inflammation, and neurotransmitter disturbances. In the liver, ethanol metabolism generates excessive free radicals that overwhelm the scavenging capacity of SOD and GSH and inhibit the Nrf2 signaling pathway, further impairing antioxidant enzyme activities, leading to the accumulation of the lipid peroxidation product MDA and damage to hepatocyte structure [27]. Meanwhile, oxidative stress disrupts fatty acid metabolism, manifested as elevated serum levels of LDL-C, TC, and TG, reflecting abnormalities in lipid transport [28]. Furthermore, ethanol damages the intestinal barrier, allowing LPS to enter the portal circulation, which activates the TLR4/NF-κB pathway in Kupffer cells, triggering the release of inflammatory cytokines such as TNF-α and IL-1, thereby exacerbating hepatocyte necrosis and fibrosis [29,30]. In the central nervous system, alcohol can directly cross the blood–brain barrier or indirectly affect the brain via peripheral organs. Ethanol metabolism within the brain generates ROS, which activate astrocytic TLR4 and IL-1R I, upregulate iNOS/NO, and amplify oxidative stress and inflammatory responses through an NF-κB positive feedback loop [31,32,33]. At the same time, it disrupts the homeostasis of AChE, GABA, 5-HT, DA, and other neurotransmitter systems, impairs signal transduction, and leads to symptoms such as intoxication and motor incoordination [34]. Consistent with this, the mice in the Model group exhibited typical features of acute liver and brain injury following excessive alcohol exposure. In the liver, serum liver enzymes and lipid levels were significantly elevated, hepatic oxidative stress and inflammatory responses were enhanced, and pathological sections revealed marked steatosis and inflammatory infiltration. In the brain, the mice displayed behavioral abnormalities such as unsteady gait, limb weakness, drunkenness and lethargy, as well as a shortened sobering-up time; furthermore, oxidative stress, inflammatory pathways, and neurotransmitter homeostasis in the brain were all disrupted. These results confirm that the acute alcoholic liver injury and brain injury models were successfully established, respectively.

Compared with the Model group, CS–SCP gel treatment prolonged the latency to intoxication, shortened the sleep maintenance time and sobering time in intoxicated mice, and exhibited a favorable anti-intoxication effect that was superior to the commercial drug SFJB. The histopathological analysis revealed that after CS–SCP gel treatment, hepatocytes were arranged regularly, with rare inflammatory infiltration, edema, and steatosis, and the semi-quantitative histological score was significantly reduced. The biochemical results show that CS–SCP gel treatment could reduce the serum levels of TG, TC, and LDL-C, the activities of ALT, AST, and ALP, and the blood ethanol concentration, while increasing SOD activity and decreasing the levels of ROS, MDA, TNF-α, and IL-1 in liver tissue. Similar results have been reported in multiple studies [35,36,37]. These findings suggest that CS–SCP gel can effectively enhance antioxidant enzyme activity, alleviate lipid peroxidation damage, and inhibit the overexpression of inflammatory cytokines. Accordingly, we hypothesize that CS–SCP gel confers a protective effect against alcoholic liver injury via modulating oxidative stress-associated signaling pathways. Meanwhile, compared with the Model group, CS–SCP gel treatment elevated SOD activity while decreasing ROS and MDA contents, thereby ameliorating neuronal lipid-peroxidation-mediated damage in mouse brain tissues. Meanwhile, it decreased toxic NO accumulation, downregulated central pro-inflammatory cytokine levels, and attenuated neuroinflammation. Similar findings have been reported by Amirshahrokhi et al. [32]. Furthermore, CS–SCP gel effectively corrected alcohol-induced neurotransmitter disturbances, restored central nervous conduction function, and ameliorated the neurological symptoms of intoxication in mice. Accordingly, we hypothesize that CS–SCP gel may maintain the balance between superoxide anions and nitric oxide (NO), thereby alleviating oxidative stress and neuroinflammation in the brain, ameliorating neurotransmitter dysregulation, and ultimately exerting a protective effect against alcohol-induced brain injury.

Meanwhile, the SCP group and the CS gel group also showed significant improvements in multiple indicators. We speculate that CS gel mainly functions through the formation of a physical barrier, gastric ethanol retention, and the intrinsic antioxidant activity of CS itself, whereas SCP primarily provides pharmacological protection through its own antioxidant and anti-inflammatory activities. Moreover, multiple indicators demonstrated that the CS–SCP gel group outperformed the CS gel and SCP groups alone, exhibiting a markedly enhanced composite protective effect, and for some indicators, the level of protection was comparable to that of the positive control silymarin. This enhanced effect can be attributed to the combined effects of multiple factors: the gel-based sustained-release system helps prolong the duration of action of SCP and increases the local effective drug concentration in the stomach, while its physical barrier effect and the pharmacological intervention of the peptide drug complement each other at both spatial and mechanistic levels.

This study preliminarily demonstrated that CS–SCP gel can effectively alleviate acute alcohol-induced liver and brain injury. However, within the scope of the present work, we primarily focused on constructing the peptide–chitosan composite hydrogel and evaluating its in vivo pharmacodynamics. Studies such as peptide sequence analysis, mechanical property testing of the hydrogel, in vitro simulated digestion assessment, and evaluation of formulation stability and release behavior in simulated gastrointestinal fluids were not conducted. These aspects will be systematically investigated as critical quality-control parameters in subsequent formulation optimization studies. Meanwhile, since the protective effects of CS–SCP gel against acute alcohol-induced liver and brain injury were mainly explored using mouse models, the discussion regarding the underlying mechanisms is based on reasonable inferences drawn from the current characterization data and existing literature. Future research will focus on validating the precise mechanisms of the relevant pathways at the cellular, protein, and gene levels, as well as conducting detailed pharmacokinetic analyses, long-term toxicity assessments, and efficacy validation, thereby laying a foundation for clinical translation.

## 4. Materials and Methods

### 4.1. Materials

SCP was supplied by Xi’an Aomengsisheng Biotechnology Co., Ltd. (Xi’an, China). Hong Xing Er Guo Tou was purchased from Beijing Red Star Co., Ltd. (Concentration of fermented alcohol: 56% vol. Beijing, China). Hematoxylin–eosin dye was supplied by Sigma-Aldrich (Shanghai, China). Chitosan (degree of deacetylation 80%) was supplied by Shanghai Macklin Biochemical Co., Ltd. (Shanghai, China). The 0.01 mol/L PBS buffer (pH 7.2–7.4) and 4% paraformaldehyde (PFA) solution were obtained from Shanghai Yeyuan Biotechnology Co., Ltd. (Shanghai, China). Silibinin capsules were obtained from Tianjin Tasly Sante Pharmaceutical Co., Ltd. (Tianjin, China). The ethanol colorimetric assay kit was supplied by Elabscience (Wuhan, China), and ELISA kits were purchased from Nanjing Jiancheng Bioengineering Institute (Nanjing, China). All the other reagents used were of analytical grade.

### 4.2. Animals

A total of 222 SPF male Kunming mice (30 ± 2 g) were purchased from the Guangdong Medical Laboratory Animal Center (Animal Production License No.: SCXK (Yue) 2022-0002. Animal Quality Certificate No.: 44007200120269). The animals were housed under controlled conditions at 23 ± 2 °C and 55 ± 10% humidity with a 12 h light/dark cycle. All the animal experiments were conducted in strict accordance with the guidelines of the Laboratory Animal Ethics Committee of Guangdong Ocean University (Approval No. GOU-LAE-2023-031). After one week of acclimatization, subsequent experiments were performed.

### 4.3. Molecular Weight Distribution and Amino Acid Composition of SCP

The molecular weight distribution and amino acid composition of SCP were determined using GPC (Waters E2695, Waters Corporation, Milford, MA, USA) and an automatic amino acid analyzer (Biochrom 30+, Biochrom Ltd., Cambridge, UK) [19].

### 4.4. Preparation of CS–SCP Gel and CS Gel

Preparation of CS–SCP gel: Accurately weigh 1.000 g of CS and 1.350 g of SCP, dissolve them in 50 mL of 1% (*v*/*v*) glacial acetic acid solution, stir to dissolve completely, and cool in an ice bath for 15 min to obtain solution A_1_. Separately, dissolve 1.050 g of NaHCO_3_ in 50 mL of ultrapure water to obtain solution B_1_. Under ice-bath conditions, slowly add solution B_1_ into solution A_1_ with stirring. After thorough mixing, the chitosan–sea cucumber peptide sol is obtained. Transfer the sol to a 37 °C constant-temperature water bath to complete the phase-transition gelation, yielding the CS–SCP gel test sample. Store at 4 °C for subsequent use.

Preparation of blank chitosan hydrogel (CS gel): The CS gel was prepared under the same processing conditions as the CS–SCP gel. Accurately weigh the same amount of CS as used in the preparation of CS–SCP gel and dissolve it in 50 mL of 1% (*v*/*v*) glacial acetic acid solution. Stir to dissolve completely, then cool in an ice bath for 15 min to obtain solution A_2_. Weigh the same amount of NaHCO_3_ as used in the preparation of CS–SCP gel and dissolve it in 50 mL of ultrapure water to obtain solution B_2_. Under ice-bath conditions, slowly add solution B_2_ into solution A_2_ with stirring. After thorough mixing, the blank chitosan sol is obtained. Transfer the sol to a 37 °C constant-temperature water bath to complete the phase-transition gelation, yielding the CS gel test sample. Store at 4 °C for subsequent use.

### 4.5. Characterization of CS–SCP Gel and CS Gel

#### 4.5.1. Macroscopic Morphology

The prepared chitosan–sea cucumber peptide sol was transferred into a vial, and its sol state was photographed. The vial was then placed in a 37 °C constant-temperature water bath to induce phase-transition gelation. After complete gelation, the sample was photographed again to compare the macroscopic morphological changes before and after gelation.

#### 4.5.2. Scanning Electron Microscopy (SEM)

The freeze-dried CS gel and CS–SCP gel samples were sputter-coated with gold and then observed and photographed under an SEM (TESCAN MIRA LMS, TESCAN, Brno, Czech Republic) at an accelerating voltage of 10 kV. The SEM images only represent the gel framework in the dried state.

#### 4.5.3. Determination of Gelling Time

The temperature-dependent rheological properties of the chitosan–sea cucumber peptide sol were characterized using a rheometer (HAAKE MARS40, Thermo Fisher Scientific, Vreden, Germany) equipped with a cone/plate measuring system (C35/1, φ = 35 mm, angle = 1°). A 1 mL aliquot of the sol sample was loaded onto the rheometer, and measurements were performed at a constant frequency of 1 Hz over a temperature range of 20–60 °C. The variations of the storage modulus (G′) and loss modulus (G″) with temperature were recorded and analyzed to determine the gel phase transition temperature and the mechanical rheological characteristics.

#### 4.5.4. Fourier Transform Infrared Spectroscopy (FTIR)

The CS, NaHCO_3_, SCP, CS–SCP gel, CS gel, and KBr in a dry powder state were compressed into tablets, and then the infrared spectra were determined with a Fourier transform infrared spectrometer (Spectrum 100, PerkinElmer, Waltham, MA, USA) with a scanning wavenumber range of 4000–400 cm^−1^ and a resolution of 4 cm^−1^. The interactions between the components and the structural characteristics of the hydrogels were analyzed.

#### 4.5.5. Water Absorption Ratio

Freeze-dried CS–SCP gel and CS gel were accurately weighed, soaked in distilled water for 30 min, then removed. The free water on the gel surface was carefully blotted with filter paper, and the gels were weighed again. All the samples were measured in triplicate, and the average values were recorded. The water absorption rate was calculated according to the following formula:

(1) $$W_{r}\;(\%)\;=\;\frac{m_{t}-m_{0}}{m_{0}}\;\times \;100\%$$

W_r_: water absorption rate; m_t_: the mass of the gel after soaking; m_0_: the initial mass of the freeze-dried gel.

#### 4.5.6. Cumulative Drug Release Rate

CS–SCP gel was put into a dialysis bag for dialysis under magnetic agitation. At predetermined time points, a 1 mL aliquot of the release medium was withdrawn, and its absorbance was measured at 217 nm using a UV spectrophotometer (UV-756 S, Shanghai Lengguang Technology Co., Ltd., Shanghai, China). The SCP concentration was determined based on an SCP standard curve (y = 5.71644x + 0.00997, R^2^ = 0.9999). After each sampling, an equal volume of fresh PBS was replenished to maintain a constant total volume of the release medium. For a peptide mixture, the absorption of peptide bonds at 217 nm cannot specifically distinguish between individual peptide components; therefore, the release profile reflects the cumulative release trend of the total peptides rather than the release behavior of a specific active peptide monomer. The amount of SCP released at each time point was calculated from the standard curve, and each experiment was performed in triplicate with the average values recorded. The cumulative release rate of the drug from the gel at time t was calculated according to the following formula:

(2) $$Q\;(\%)\;=\;\frac{C_{ti}V_{0}+V_{e}\sum C_{t}(i-1)}{m}\times 100\%$$

Q: cumulative drug release rate (%); C_ti_: concentration of SCP released from the gel at time t; V_0_: total volume of release medium (mL); V_e_: the sample volume; i: number of times to replace release medium; m: total amount of SCP loaded in the gel.

### 4.6. Animal Experiments

#### 4.6.1. Preparation of the Test Substance

a. Positive Control Drug Silibinin Solution (SFJB): The powder from silibinin capsules was ground into a fine powder in a mortar. An accurately weighed 70 mg portion was dissolved in distilled water, and the volume was adjusted to 10 mL. The solution was stored at 4 °C for subsequent use.

b. SCP Solution: An accurately weighed 135 mg sample of SCP was dissolved in distilled water, adjusted to 10 mL, and stored at 4 °C until use.

c. CS–SCP gel and CS gel were prepared as described in Section 4.4.

#### 4.6.2. The Optimal Intoxicating Dose in Mice

Thirty mice described in Section 4.2 were randomly divided into three groups (*n* = 10 per group). After fasting for 12 h with free access to water, each group was intragastrically administered 56% (*v*/*v*) Hongxing Erguotou liquor at doses of 16, 17, and 18 mL/kg body weight, respectively. The mice were observed for signs of intoxication, and the numbers of intoxicated and dead mice were recorded. The dose that resulted in the highest intoxication rate and the lowest mortality was selected as the optimal dose for establishing subsequent models of anti-inebriation, sobering, and acute alcohol-induced injury.

In this experiment, the loss and recovery of the righting reflex in mice were used as the indicators of intoxication and sobering. The times of administration, intoxication, and recovery were recorded. The latency to intoxication, sleep maintenance time, and sobering time were calculated, respectively. The formulas are as follows:

T_l_ = T_i_ − T_a_ (3)

T_m_ = T_r_ − T_i_ (4)

T_s_ = T_l_ + T_m_ (5)

T_l_: The latency to intoxication; T_i_: the times of intoxication; T_a_: the times of administration; T_m_: sleep maintenance time; T_r_: the times of recovery; T_s_: sobering time.

#### 4.6.3. Anti-Intoxication Test in Mice

Sixty mice described in Section 4.2 were randomly divided into five groups (*n* = 12): Model group, silibinin positive control group (SFJB group), sea cucumber peptide sol group (SCP group), blank chitosan hydrogel group (CS gel group), and thermosensitive CS–SCP hydrogel group (CS–SCP gel group). Each group was intragastrically administered the corresponding test substance at a dose of 10 mL/kg (i.e., 0.1 mL/10 g body weight), while the Model group received an equivalent volume of distilled water. One hour later, all the mice were intragastrically administered 56% (*v*/*v*) Hongxing Erguotou liquor at the optimal intoxicating dose determined in Section 4.6.2. The latency to intoxication, sleep maintenance time, and sobering time were measured and calculated for each group to evaluate and compare the anti-intoxication effects of the test substances.

#### 4.6.4. Blood Ethanol Concentration in Mice

Sixty mice described in Section 4.2 were randomly divided into five groups (*n* = 12). The grouping, pretreatment dosing regimen, and modeling protocol were identical to those of the anti-intoxication test described in Section 4.6.3. At 0.5, 1, 2, and 3 h after alcohol gavage, three mice were randomly selected from each group for retro-orbital blood collection. The blood ethanol concentration at each time point was measured and calculated strictly following the kit manufacturer’s instructions.

#### 4.6.5. Acute Alcohol-Induced Injury Modeling in Mice

Seventy-two mice described in Section 4.2 were randomly divided into six groups (*n* = 12): Normal group, Model group, SFJB group, SCP group, CS gel group, and CS–SCP gel group. The treatment groups received the corresponding test substance intragastrically at a dose of 10 mL/kg (0.1 mL/10 g body weight) once daily, while the Normal and Model groups received an equivalent volume of distilled water for 7 consecutive days. One hour after the final administration, all the groups except the Normal group were intragastrically administered 56% (*v*/*v*) Hongxing Erguotou liquor at the optimal intoxicating dose determined in Section 4.6.2 to induce acute alcohol-induced injury. The Normal group received an equivalent volume of distilled water instead. Samples were collected at predetermined time points after modeling.

#### 4.6.6. Serum Collection and Measurement of Liver Function and Blood Lipid Parameters

Six hours after the completion of the animal modeling described in Section 4.6.5, six mice were randomly selected from each group, and blood was collected from the retro-orbital venous plexus (approximately 0.4 mL per mouse). The fresh blood was centrifuged at 3000 rpm for 15 min at 4 °C, and the supernatant serum was collected. The levels of ALT, AST, ALP, TC, TG, and LDL-C were determined strictly following the kit manufacturer’s instructions.

#### 4.6.7. Brain Tissue Homogenate Preparation and Measurement of Related Parameters

The remaining mice in each group (*n* = 6) described in Section 4.6.6 were sacrificed by cervical dislocation. The brain tissues were rapidly removed, rinsed with pre-chilled saline, blotted dry with filter paper, and accurately weighed. Cold PBS was added at a weight (g) to PBS buffer (mL) ratio of 1:9, and the tissue was homogenized using a cryogenic tissue grinder (JXFSTPRP-CL, Shanghai Jingxin Industrial Development Co., Ltd., Shanghai, China) to prepare a 10% brain tissue homogenate. The homogenate was centrifuged at 2500 rpm for 10 min at 4 °C, and the supernatant was collected. The levels of ROS and the activities of SOD and AChE, as well as the levels of MDA, TNF-α, IL-1β, IL-6, NO, iNOS, GABA, 5-HT, DA, and NF-κB were determined according to the kit manufacturer’s instructions.

#### 4.6.8. Histopathological Analysis of Liver Tissue in Mice

After brain tissue collection as described in Section 4.6.7, the liver tissues were rapidly removed. Small pieces of the left lobe of each liver were quickly excised and fixed in 4% paraformaldehyde for histological observation, and the remaining liver tissues were stored for later use.

The fixed liver tissues were then dehydrated through a graded ethanol series, embedded in paraffin, and sectioned at 5 μm thickness. The sections were stained with hematoxylin and eosin (H&E) and observed under a light microscope to evaluate cellular morphology, inflammatory infiltration, and lipid deposition in the liver tissues of each group. Images were captured for analysis.

The samples were examined by an experienced pathologist who was blinded to the experimental group allocation, and the severity of the pathological changes was graded according to the histopathological scoring criteria proposed by Maneerat and colleagues [38], as described below. A total of six sections were evaluated per group.

The scoring criteria for hepatic steatosis were as follows:

0 = less than 5% of hepatocytes containing fat

1 = 5–33% of hepatocytes containing fat

2 = 34–66% of hepatocytes containing fat

3 = more than 66% of hepatocytes containing fat

The scoring criteria for lobular inflammation were as follows:

0 = none

1 = fewer than 2 inflammatory foci per 200× field

2 = 2–4 inflammatory foci per 200× field

3 = more than 4 inflammatory foci per 200× field

The scoring criteria for hepatocellular ballooning were as follows:

0 = none

1 = slight ballooning

2 = prominent ballooning

#### 4.6.9. Liver Tissue Homogenate Preparation and Measurement of Related Parameters

The stored liver tissues from Section 4.6.8 were used. Approximately 0.5 g of liver tissue per mouse was homogenized in pre-chilled saline at a weight (g) to saline volume (mL) ratio of 1:9 using a cryogenic tissue grinder to prepare a 10% liver tissue homogenate. The homogenate was centrifuged at 3000 rpm for 10 min at 4 °C, and the supernatant was collected. The levels of ROS, MDA, NF-κB, TNF-α, and IL-1, as well as the activity of SOD were determined according to the kit manufacturer’s instructions.

#### 4.6.10. Statistical Analysis

GraphPad Prism 10.6.0 software was used for all the statistical analyses. Data were expressed as mean ± standard deviation (SD). One-way analysis of variance (ANOVA) followed by Dunnett’s post hoc test was used to evaluate significant differences among the groups. Statistical significance was defined as *p* < 0.05.

## Figures and Tables

**Table 1:** The molecular weight distribution of SCP.

Molecular Weight Range (Da)	Weight Average Molecular Weight (Da)	Proportion (%)
>5000	5670	3.67
5000–4000	4049	11.51
4000–3000	3035	26.70
3000–2000	2760	32.52
<2000	1650	25.60

**Table 2:** Composition and content of the amino acids in SCP.

Amino Acid	Retention Time (min)	Peak Area (mV·s)	Contents (g/100 g)	Proportion (%)
Asp	7.399	1132.153	8.1408	8.453
Thr	9.203	608.282	3.8784	4.027
Ser	10.077	886.453	4.558	4.733
Glu	11.608	1644.683	10.6346	11.043
Pro	13.264	906.49	11.0802	11.506
Gly	17.320	4910.795	15.5903	16.189
Ala	18.091	2097.854	8.9415	9.285
Cys-Cys	21.824	60.031	0.513	0.533
Val	21.969	398.854	2.6509	2.753
Met	23.908	628.121	3.9501	4.102
Ile	25.245	651.085	4.0314	4.186
Leu	26.377	870.521	4.9686	5.159
Tyr	29.579	142.910	1.3188	1.369
Phe	30.457	285.779	2.2651	2.352
His	35.744	195.326	1.5265	1.585
Lys	36.789	664.053	4.9892	5.181
Arg	45.449	1011.204	7.2648	7.544

**Table 3:** Drunkenness rate, death rate, drunkenness latency, sleep maintenance time and sobering time of mice in different groups ($\overset{-}{x}$ ± SD, *n* = 12).

Group	Intoxication Rates (%)	Mortality Rates (%)	Latency to Intoxication (min)	Sleep Maintenance Time (min)	Sobering Time (min)
Model	91.67	50	60.80 ± 41.41	673.60 ± 80.19	734.40 ± 55.20
SFJB	83.33	41.67	80.00 ± 41.54	580.60 ± 141.12	660.60 ± 111.86
SCP	75.00	16.67	91.17 ± 85.17	556.17 ± 106.54	647.33 ± 55.36
CS gel	75.00	8	67.25 ± 51.95	479.13 ± 151.62	546.38 ± 122.84
CS–SCP gel	58.33	0	118.14 ± 49.03	310.00 ± 160.46 **	428.14 ± 159.24 **

** *p* < 0.01 vs. Model group.

## Data Availability

The data that support the findings of this study are available from the corresponding author upon reasonable request.

## Abbreviations

The following abbreviations are used in this manuscript:

CS	Chitosan
SCP	Sea cucumber peptides
CS–SCP gel	Chitosan–sea cucumber peptides hydrogel
CAT	Catalase
GSH-Px	Glutathione peroxidase
TNF-α	Tumor necrosis factor-α
SOD	Superoxide dismutase
ALP	Alkaline phosphatase
ALT	Alanine aminotransferase
AST	Aspartate aminotransferase
LDL-C	Low-density lipoprotein cholesterol
TC	Total cholesterol
TG	Triglycerides
ROS	Reactive oxygen species
MDA	Malondialdehyde
IL-1	Interleukin-1
AChE	Acetylcholinesterase
GABA	γ-aminobutyric acid
5-HT	5-hydroxytryptamine
DA	Dopamine
IL-6	Interleukin-6
IL-1β	Interleukin-1β
iNOS	Inducible nitric oxide synthase
NO	Nitric oxide
SFJB	Silibinin capsules
AUC	The area under the curve

## References

[1] World Health Organization (2024). Global Status Report on Alcohol and Health and Treatment of Substance Use Disorders.

[2] Li J., Liu Y., Chen X., Luo M., Yin M., Xie X., Ai Y., Zhang X., He J. (2024). Therapeutic potential of Lingjiao Gouteng decoction in acute alcohol intoxication and alcohol-induced brain injury involving the RhoA/ROCK2/NF-kappaB signaling pathway. J. Ethnopharmacol..

[3] Lee J., Lee J.Y., Kang H. (2025). Excessive alcohol consumption: A driver of metabolic dysfunction and inflammation. Front. Toxicol..

[4] D’Angelo A., Petrella C., Greco A., Ralli M., Vitali M., Giovagnoli R., De Persis S., Fiore M., Ceccanti M., Messina M.P. (2022). Acute alcohol intoxication: A clinical overview. Clin. Ter..

[5] Wang Q., Shi J., Zhong H., Abdullah, Zhuang J., Zhang J., Wang J., Zhang X., Feng F. (2021). High-degree hydrolysis sea cucumber peptides improve exercise performance and exert antifatigue effect via activating the NRF2 and AMPK signaling pathways in mice. J. Funct. Foods.

[6] Wan H., Han J., Tang S., Bao W., Lu C., Zhou J., Ming T., Li Y., Su X. (2020). Comparisons of protective effects between two sea cucumber hydrolysates against diet induced hyperuricemia and renal inflammation in mice. Food Funct..

[7] Zhu B.-W., Dong X.-P., Zhou D.-Y., Gao Y., Yang J.-F., Li D.-M., Zhao X.-K., Ren T.-T., Ye W.-X., Tan H. (2012). Physicochemical properties and radical scavenging capacities of pepsin-solubilized collagen from sea cucumber *Stichopus japonicus*. Food Hydrocoll..

[8] Zhu Q., Zhuo H., Yang L., Ouyang H., Chen J., Liu B., Huang H. (2022). A Peptide HEPFYGNEGALR from *Apostichopus japonicus* Alleviates Acute Alcoholic Liver Injury by Enhancing Antioxidant Response in Male C57BL/6J Mice. Molecules.

[9] Hao S., Wang Y., Lv Y., Liu K., Tan M. (2026). A hepatocyte targeted silymarin sea cucumber peptide-galactose nanoparticle for the alleviation of ethanol-induced damage in cells. Colloids Surf. B Biointerfaces.

[10] Wang X., Yang Z., Zhang W., Xing L., Luo R., Cao S. (2024). Obstacles, research progress, and prospects of oral delivery of bioactive peptides: A comprehensive review. Front. Nutr..

[11] Wang X., Yang Z., Zhang W., Xing L., Luo R., Cao S. (2026). Translational Advances in Chitosan Biomaterials: From Molecular Modification to Clinical Medicine. ACS Omega.

[12] Hilitanu L.N., Mititelu-Tarțău L., Bogdan M., Buca B.R., Păuna A.-M.R., Pavel L.L., Pelin A.-M., Meca A.-D., Popa G.E. (2022). The Use of Chitosan-Coated Nanovesicles in Repairing Alcohol-Induced Damage of Liver Cells in Mice. Medicina.

[13] Mura P., Maestrelli F., Cirri M., Mennini N. (2022). Multiple Roles of Chitosan in Mucosal Drug Delivery: An Updated Review. Mar. Drugs.

[14] Lu S., Zhang L., Hu Z., Kong S., Zhang Z., Li G. (2021). Optimized preparation of gastric acid-response sulfhydryl functionalized chitosan/alginate/tilapia peptide hydrogel and its protective effects on alcohol-induced liver and brain injury. RSC Adv..

[15] Taokaew S., Kaewkong W., Kriangkrai W. (2023). Recent Development of Functional Chitosan-Based Hydrogels for Pharmaceutical and Biomedical Applications. Gels.

[16] Pieklarz K., Galita G., Tylman M., Maniukiewicz W., Kucharska E., Majsterek I., Modrzejewska Z. (2021). Physico-Chemical Properties and Biocompatibility of Thermosensitive Chitosan Lactate and Chitosan Chloride Hydrogels Developed for Tissue Engineering Application. J. Funct. Biomater..

[17] Liu L., Tang X., Wang Y., Guo S. (2011). Smart gelation of chitosan solution in the presence of NaHCO_3_ for injectable drug delivery system. Int. J. Pharm..

[18] Stealey S.T., Gaharwar A.K., Zustiak S.P. (2023). Laponite-Based Nanocomposite Hydrogels for Drug Delivery Applications. Pharmaceuticals.

[19] Yu Y., Wu G., Jiang Y., Li B., Feng C., Ge Y., Le H., Jiang L., Liu H., Shi Y. (2020). Sea Cucumber Peptides Improved the Mitochondrial Capacity of Mice: A Potential Mechanism to Enhance Gluconeogenesis and Fat Catabolism during Exercise for Improved Antifatigue Property. Oxid. Med. Cell Longev..

[20] Park S.Y., Fernando I.P.S., Han E.J., Kim M.J., Jung K., Kang D.-S., Ahn C.-B., Ahn G. (2019). In Vivo Hepatoprotective Effects of a Peptide Fraction from Krill Protein Hydrolysates against Alcohol-Induced Oxidative Damage. Mar. Drugs.

[21] Thakur S., Govender P.P., Mamo M.A., Tamulevicius S., Thakur V.K. (2017). Recent progress in gelatin hydrogel nanocomposites for water purification and beyond. Vacuum.

[22] Lee J.Y., Jee Y.-M., Yang K., Ryu T. (2025). Alcohol-Induced Oxidative Stress and Gut-Liver-Brain Crosstalk: Expanding the Paradigm from ALD to MetALD. Antioxidants.

[23] Wang R., Xu S., Zhang M., Feng W., Wang C., Qiu X., Li J., Zhao W. (2024). Multifunctional chitosan-based hydrogels loaded with iridium nanoenzymes for skin wound repair. Carbohydr. Polym..

[24] Zhou W., Zhu L., Zu X., Ma Y., Shen P., Hu Y., Zhang P., Ni Z., Wang N., Sun D. (2025). A novel antioxidant and anti-inflammatory carboxymethylcellulose/chitosan hydrogel loaded with cannabidiol promotes the healing of radiation-combined wound skin injury in the (60)Co gamma-irradiated mice. Phytomedicine.

[25] Xing C., Hou L., Sun C., Chen H., Li Y., Li L., Wu Y., Li L., An H., Wen Y. (2025). Injectable polypeptide/chitosan hydrogel with loaded stem cells and rapid gelation promoting angiogenesis for diabetic wound healing. Int. J. Biol. Macromol..

[26] Tong J., Zhou H., Zhou J., Chen Y., Shi J., Zhang J., Liang X., Du T. (2022). Design and evaluation of chitosan-amino acid thermosensitive hydrogel. Mar. Life Sci. Technol..

[27] Yan C., Hu W., Tu J., Li J., Liang Q., Han S. (2023). Pathogenic mechanisms and regulatory factors involved in alcoholic liver disease. J. Transl. Med..

[28] Xu Q., Guo M., Wang H., Liu H., Wei Y., Wang X., Mackay C.R., Wang Q. (2024). A Butyrate-Yielding Dietary Supplement Prevents Acute Alcoholic Liver Injury by Modulating Nrf2-Mediated Hepatic Oxidative Stress and Gut Microbiota. Int. J. Mol. Sci..

[29] Rungratanawanich W., Lin Y., Wang X., Kawamoto T., Chidambaram S.B., Song B.-J. (2023). ALDH2 deficiency increases susceptibility to binge alcohol-induced gut leakiness, endotoxemia, and acute liver injury in mice through the gut-liver axis. Redox Biol..

[30] Shen H., Liangpunsakul S., Iwakiri Y., Szabo G., Wang H. (2025). Immunological mechanisms and emerging therapeutic targets in alcohol-associated liver disease. Cell. Mol. Immunol..

[31] Ray B., Rungratanawanich W., LeFort K.R., Chidambaram S.B., Song B.-J. (2024). Mitochondrial Aldehyde Dehydrogenase 2 (ALDH2) Protects against Binge Alcohol-Mediated Gut and Brain Injury. Cells.

[32] Amirshahrokhi K., Niapour A. (2022). Methylsulfonylmethane protects against ethanol-induced brain injury in mice through the inhibition of oxidative stress, proinflammatory mediators and apoptotic cell death. Int. Immunopharmacol..

[33] Anand S.K., Ahmad M.H., Sahu M.R., Subba R., Mondal A.C. (2023). Detrimental Effects of Alcohol-Induced Inflammation on Brain Health: From Neurogenesis to Neurodegeneration. Cell Mol. Neurobiol..

[34] Abrahao K.P., Salinas A.G., Lovinger D.M. (2017). Alcohol and the Brain: Neuronal Molecular Targets, Synapses, and Circuits. Neuron.

[35] He W., Zhang B., Li S., Qian Y. (2026). Quercetin attenuates acute alcohol-induced liver injury in mice by modulating lipid metabolism, oxidative stress, and inflammation. Front. Pharmacol..

[36] Lai W., Zhou S., Bai Y., Che Q., Cao H., Guo J., Su Z. (2024). Glucosamine attenuates alcohol-induced acute liver injury via inhibiting oxidative stress and inflammation. Curr. Res. Food Sci..

[37] Tang X., Wei R., Deng A., Lei T. (2017). Protective Effects of Ethanolic Extracts from Artichoke, an Edible Herbal Medicine, against Acute Alcohol-Induced Liver Injury in Mice. Nutrients.

[38] Chayanupatkul M., Somanawat K., Chuaypen N., Klaikeaw N., Wanpiyarat N., Siriviriyakul P., Tumwasorn S., Werawatganon D. (2022). Probiotics and their beneficial effects on alcohol-induced liver injury in a rat model: The role of fecal microbiota. BMC Complement. Med. Ther..

